# Genotypic and phenotypic diversity of *Prototheca spp.* recovered from bovine mastitis in terms of antimicrobial resistance and biofilm formation ability

**DOI:** 10.1186/s12917-022-03546-4

**Published:** 2022-12-26

**Authors:** Niloufar Tashakkori, Hamideh Kalateh Rahmani, Babak Khoramian

**Affiliations:** 1 Department of Clinical Sciences, Faculty of Veterinary Medicine, Ferdowsi University of Mashhad, Azadi Sq., P.O. Box: 9177948974, Mashhad, Khorasan Razavi Iran; 2 Department of Pathobiology, Faculty of Veterinary Medicine, Ferdowsi University of Mashhad, Mashhad, Iran

**Keywords:** *Prototheca*, Molecular typing, RAPD-PCR, Biofilm, Antimicrobial, Antifungal

## Abstract

**Background:**

The *Prototheca* algae have recently emerged as an important cause of bovine mastitis globally. Isolates from bovine mastitis in several countries were nearly all identified as *P. bovis*, suggesting that it was the main causative agent of bovine protothecal mastitis**.** The aim of the present study was to evaluate the presence and isolation of *Prototheca spp.* in dairy farms, detect the genetic diversity among strains, determine the capacity of producing biofilm and their resistance to antifungal and antimicrobial drugs.

**Results:**

A total of 48 *Prototheca* isolates from four different farms were randomly selected to be investigated. Multiplex PCR showed all isolated colonies were *Prototheca bovis*. Performing RAPD-PCR by using OPA-4 primer, it was revealed that there was a clear amplification pattern. Different levels of biofilm production were observed among strains. Among 48 isolates, only 4 of them (8.33%) showed strong biofilm production. By using E-test strips, amphotericin B was able to inhibit the growth of all the strains tested. Disc diffusion method used for antimicrobial sensitivity test showed that the highest activity was demonstrated by gentamicin and colistin with 95.83% (46/48) and 89.58% (43/48) of sensitive strains, respectively.

**Conclusions:**

The present study showed that RAPD-PCR was a rapid tool for discriminating *P. bovis* strains. Also, gentamicin and colistin can be considered as potential antimicrobial drugs which can prevent the growth of the mentioned strains in vitro, although there is no effective clinical treatment yet. Further studies are needed in order to detect an effective clinical therapy considering biofilm production by *Prototheca spp.* and their probable role in *Prototheca* pathogenicity.

## Background

The genus *Prototheca* consists of algae ubiquitous in the environment and animal intestines [1–4]. *P. bovis* and *P. blashkae* cause chronic mastitis in cattle, resulting in severe economic losses [5], which are incurred either directly through reduced milk production, secretion of thin, watery milk containing white flakes and premature culling of affected animals or indirectly via treatment and veterinary care expenses [6, 7].

In the past, *P. zopfii* was classified into 2 genotypes (genotype 1 and 2) based on genetic assays [8]. Isolates from bovine mastitis in Germany, Italy, Japan, Portugal and Poland were nearly all identified as *P. zopfii* genotype 2 which is now named “*P. bovis*” [9], suggesting that it was the main causative agent of bovine protothecal mastitis [10, 11]. The frequency of bovine protothecal mastitis has been alarmingly increasing worldwide [5], which may represent a serious problem due to the inherent resistance of these microalgae to different classes of antimicrobial drugs [3].

Multiple intramammary treatments, milker hygiene, and milking equipment performance are all considered as risk factors for protothecal mastitis [4]. Jagielski et al., investigated the prevalence of *Prototheca* species on dairy farms in Poland and estimated the cow-level prevalence of mastitis due to *Prototheca spp.* which was 8.3% within 16 herds [7].

A positive result in bacteriologic culture tests or molecular analysis of milk samples has generally been used to identify *Prototheca spp.* as the causative agent of mastitis [12]. In order to determine any genetic diversity among *Prototheca* strains, PCR-assays (including RAPD-PCR) and RFLP assay were shown to be helpful [13].

*Prototheca spp.* are susceptible in vitro to some antimicrobials such as amphotericin B [5]. *Prototheca* biofilm structure, like bacterial biofilms, allows the pathogen to run from host immune response and effective levels of antimicrobials. Moreover, the ability of biofilm formation is likely related to chronic and hard-to-treat types of mastitis [5].

There is currently no effective therapy for *Prototheca*-induced bovine mastitis, and current infection control strategies in infected herds involve drying all the infected quarters and/or culling infected cows to manage infected herds [12].

The aim of the present study was to evaluate the presence and isolation of *Prototheca spp*. in dairy farms, detect genetic diversity among strains, determine the ability of producing biofilm and their resistance to antifungal and antimicrobial drugs.

## Results

### Identification and characterization of *Prototheca* strains

A total of 48 *Prototheca* isolates (29 from farm A [Spring 2018-Summer 2019], 4 from farm B [Spring 2018-Winter 2019], 13 from farm C [Spring 2018-Winter 2019] and 2 from farm D [Winter 2018]) were randomly selected to be investigated more. For the identification of isolates, multiplex PCR was used; all isolated colonies were confirmed to be *Prototheca bovis.*

### RAPD-PCR

**Fig. 1:**
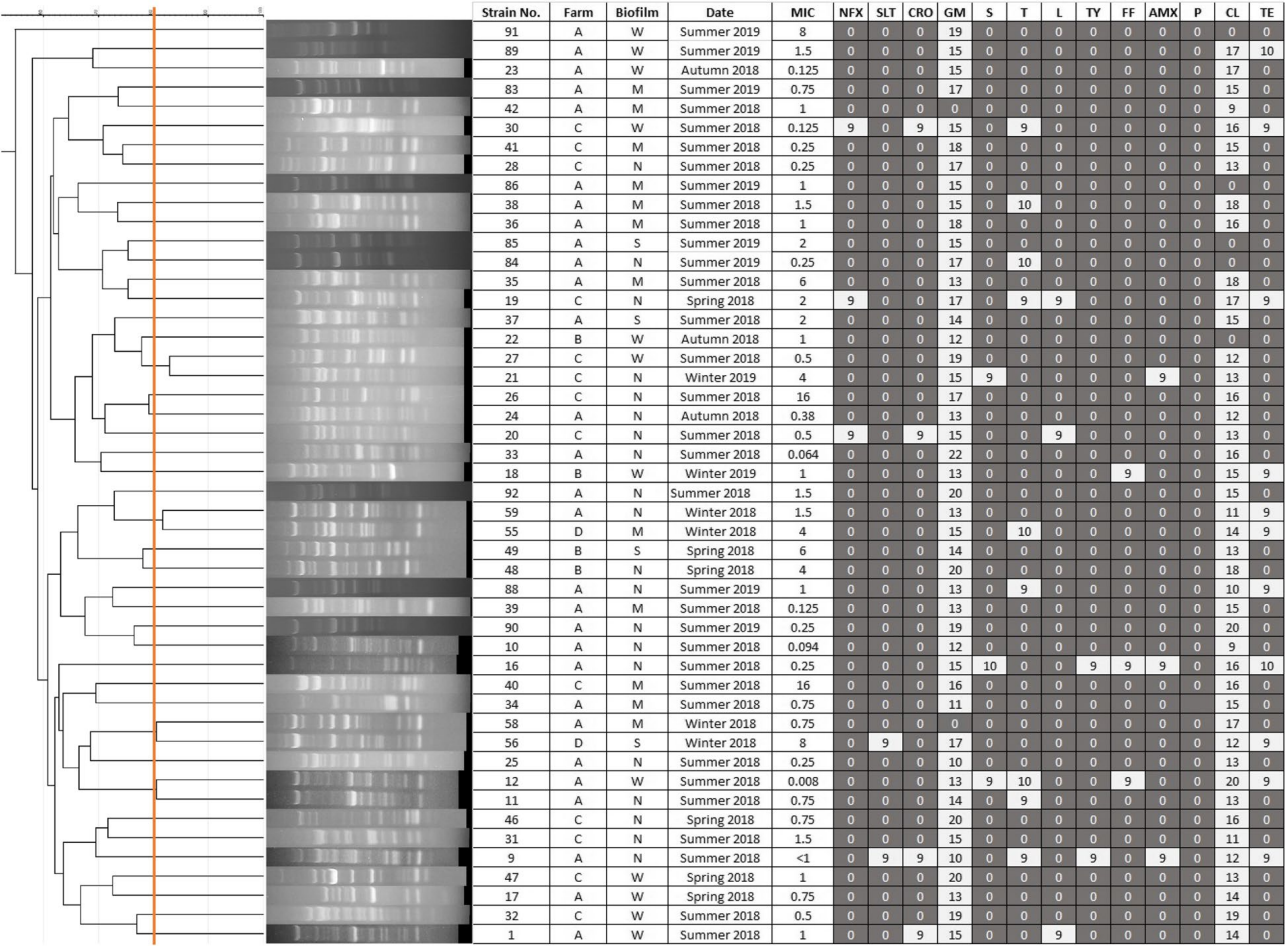
Strains divided by genetic differences shown by dendrogram, farm and season of isolation, MIC, antibiotic susceptibility, biofilm formation capacity. W: weak biofilm producer. M: medium biofilm producer. S: strong biofilm producer. N: negative. MIC: Minimum Inhibitory Concentration. NFX: Enrofloxacin, SLT: Sulfonamide + Trimethoprim, CRO: ceftriaxone, GM: gentamicin, S: streptomycin, T: oxytetracycline, L: lincomycin, TY: tylosin, FF: florfenicol, AMX: amoxicillin, P: penicillin, CL: colistin, TE: tetracycline

With regard to the results of RAPD-PCR assay, “OPA-4” primer revealed a clear amplification pattern and was consequently used for genotypic characterization of strains; while “OPA-18” primer was unable to show any noticeable diversity among strains. Figure 1 shows the dendrogram derived from amplification profile obtained from using OPA-4 primer. Dendrogram and analysis of RAPD profile of strains were carried out by using GelJ software. Strains with a similarity coefficient equal to or higher than 80% may be considered to be extremely close genotypically, as 4 pairs of strains were genotypically similar to each other. However, by considering similarity coefficient equal to 60%, only 3 pairs of strains were not similar to each other and other strains were genotypically similar.

### Biofilm formation

Different levels of biofilm production were observed among *P. bovis* strains. Among 48 isolates, 4 of them (8.33%) showed strong biofilm production (S), 12 (25%) showed moderate production (M), 12 (25%) showed weak biofilm production (W) and 20 (41.66%) of them could not produce biofilm (N). Statistical analysis using Chi square test showed that there was not a significant correlation among biofilm production ability of isolates in different farms (*p* > 0.05).

### Minimum Inhibitory Concentration (MIC)

**Fig. 2:**
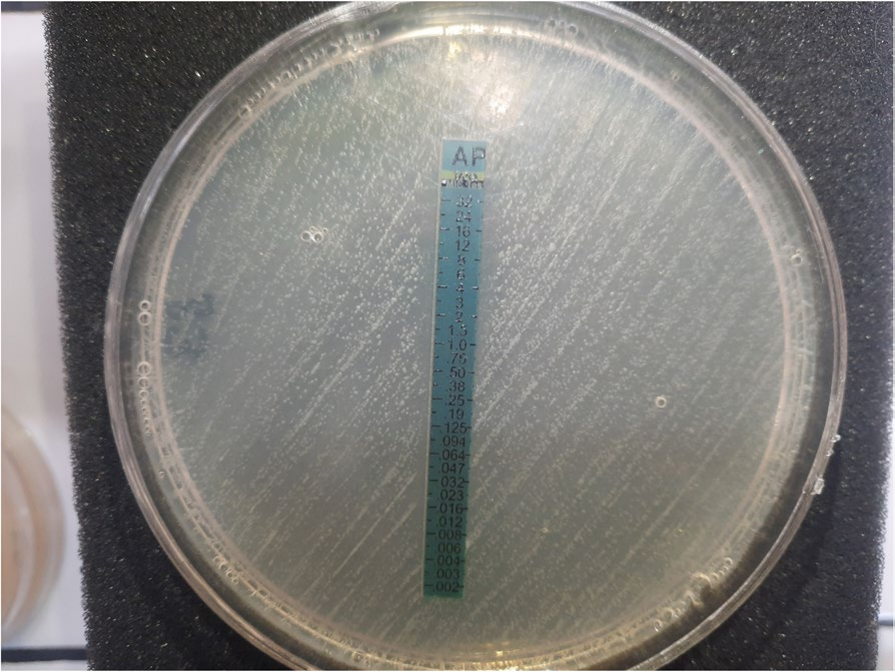
MIC using Amphotericin B strip and symmetrical inhibition ellipse centered along the strip

Amphotericin B was able to inhibit the growth of all 48 strains. The concentration of amphotericin B which was able to produce the ellipse zone in the scale of µg/ml is shown in Fig. 2. The lowest concentration of amphotericin B which could inhibit the growth of *Prototheca bovis* strain (number 12) was 0.008 µg/ml, which produced the biggest inhibition zone and the highest concentration of amphotericin B which was able to inhibit the growth of *Prototheca bovis* strains (number 26 and 40) was 16 µg/ml which produced the least inhibition zone. Kruskal–Wallis test showed a statistically significant difference among MIC values in different seasons and farms (*P* < 0.001). MIC of amphotericin B in isolates collected in years 2018 and 2019 was 0.75 and 1, respectively that showed a statistically significant difference (*P* < 0.001). The relation between MIC values and the ability of biofilm formation was significant using Kruskal–Wallis test (*p* < 0.001).

### Antimicrobial susceptibility

**Fig. 3:**
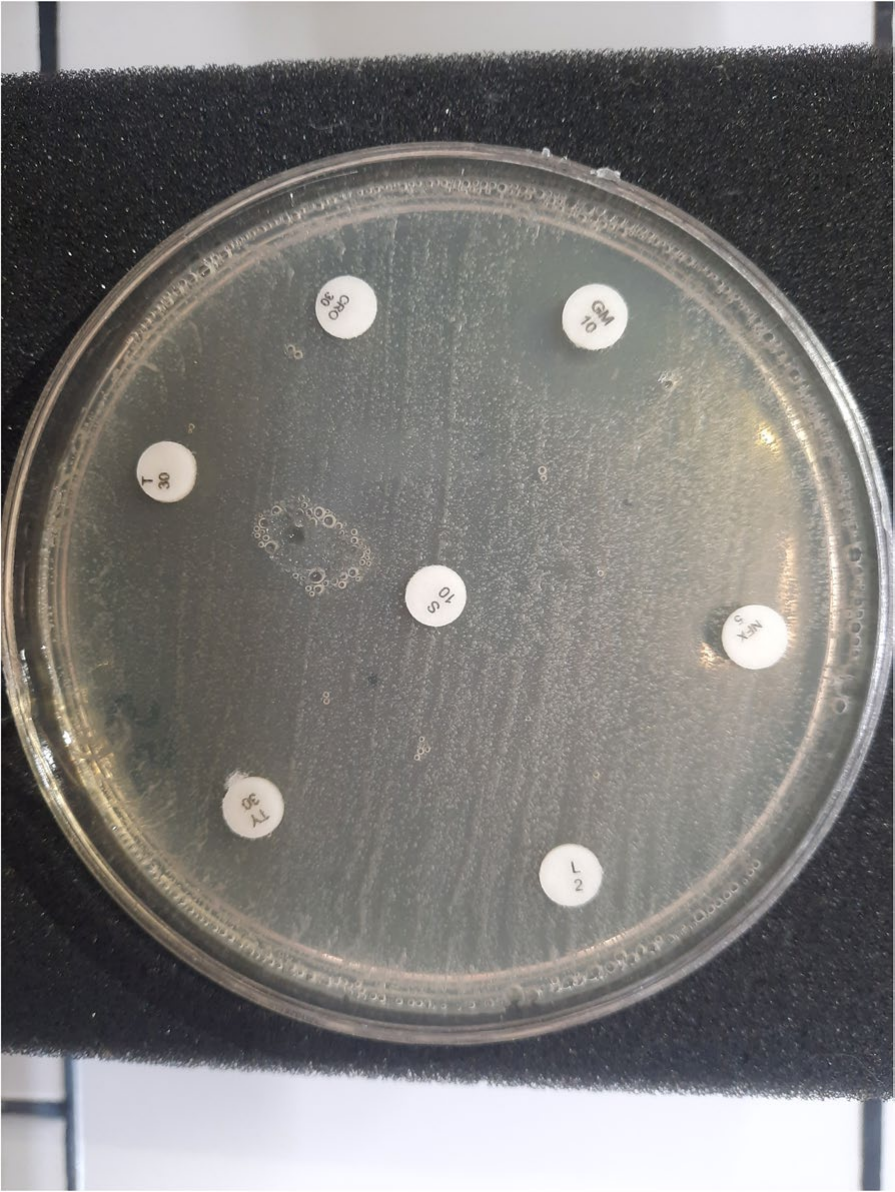
Antibiotic susceptibility test using disc diffusion method performed on Mueller Hinton agar

In vitro antibiotic susceptibility of all 48 *Prototheca* strains was examined using agar disc diffusion method with 13 different agents (Fig. 3); the results are shown in Fig. 1, in which cells with white background are demonstrating “sensitive” state and black ones are representative of “resistant” state; there was no “intermediate” state between all used antibiotics. All strains were in vitro resistant to penicillin which could not produce any inhibition zone (diameter of inhibition = 0). The lowest resistance was demonstrated by gentamicin and colistin with 95.83% (46/48) and 89.58% (43/48) of sensitive strains, respectively. Moreover, resistance to enrofloxacin (93.75% of resistant strains), Sulfonamide + Trimethoprim (95.83%), ceftriaxone (91.66%), streptomycin (93.75%), oxytetracycline (81.25%), lincomycin (93.75%), tylosin (95.83%), florfenicol (93.75%), amoxicillin (93.75%) and tetracycline (77.08% of resistant strains) was observed. There were 6 strains which were sensitive to only 1 antimicrobial, 26 strains sensitive to 2 antimicrobials, 4 strains sensitive to 3 antimicrobials, 6 strains sensitive to 4 antimicrobials, 1 strain (number 20) sensitive to 5 antimicrobials, 3 strains sensitive to 6 antimicrobials, only 1 strain (number 16) sensitive to 7 antimicrobials and another one (number 9) sensitive to 8 antimicrobials.

### Results achieved from each farm

The number of strains producing strong biofilm were 2 in farm A, 1 strain in farm B and 1 strain in farm D; there was no strain producing strong biofilm in farm C. The highest MIC (16 µg/ml) was seen in farm C and the lowest one (0.008 µg/ml) was observed in farm A. There was only 1 strain (number 9) which was sensitive to 8 antimicrobial drugs; the last-mentioned strain was isolated from farm A in summer, 2018 and was not able to produce biofilm. All *Prototheca* strains were sensitive to gentamicin except two strains which both were collected from farm A.

## Discussion

Based on the results of the present study, it has to be assumed that bovine protothecal mastitis is mainly caused by *P. bovis* as mentioned by other authors [7, 8, 13–20]. As it was stated *Prototheca zopfii* genotype 1 played no role in bovine mastitis, it was not isolated and detected among the whole samples.

RAPD PCR method is frequently chosen for its suitability as a molecular diagnostic tool for high throughput of isolates [8, 21, 22]. Morandi et al., data confirmed that RAPD-PCR gave reproducible band patterns that could be used for characterizing *Prototheca* strains at the genetic level [8].

The ability of *Prototheca spp.* to form biofilms in vitro in a classic microwell assay was performed which has been essential for the research on fungal and bacterial biofilms [5]. ﻿Kwiecinski et al., used ﻿three *Prototheca* species (﻿*P. zopfii*, *﻿P. blaschkeae* and ﻿*P. wickerhamii*) which were isolated ﻿from cattle manure, human nail infection and ﻿infected human skin. ﻿Biofilm formation was most ﻿pronounced in *P. wickerhamii*, weaker and slower in *P. blaschkeae* and *P. zopfii* [5]. Morandi et al. assessed the ability of *Prototheca zopfii* ﻿genotype 2 strains to produce biofilm [8]. Unlike the present study which detected 8.33% of strains as strong biofilm producers, the majority of isolates in Morandi et al., study could strongly produce biofilm (37/42; 88%). ﻿Gonçalves et al., ﻿determined the capacity of the *Prototheca spp*. isolates (*n* = 10) to produce biofilm. In agreement with the present study, ﻿all *P. bovis* isolates had the capacity to produce biofilm as assessed by the polystyrene microplate assay method. ﻿The results of the present study showed the ability of *P. bovis* to produce biofilm; however, further studies on gene expression are required to identify the specific genes associated with biofilm production by *P. bovis.*

All *P. bovis* strains tested in the present study were sensitive to amphotericin B in vitro due to the prevention of their growth. Morandi et al. [8] considered the geographical origin of strains and investigated MIC for different antimicrobials, except amphotericin B. They noted that *P. zopfii* genotype 2 isolated from Italy showed lower MIC values than strains from Brazil [8]. Marques et al., evaluated the susceptibility of bovine isolates of *P. wickerhamii* and *P. zopfii* to amphotericin B and nystatin by reporting MIC with the microdilution method; Nystatin showed more efficacy than amphotericin B in inhibiting *P. wickerhamii* growth; while growth inhibition of *P. zopfii* was similar for both antifungal agents [23]. However, the authors did not mention the genotype of *P. zopfii* strains used in the study. Tyczkowska-Sieron et al., studied 20 strains of *P. zopfii* isolated from milk samples taken from cows with clinical or subclinical mastitis and reported the resistance of strains to antifungal agents such as amphotericin B using MIC technique; all strains showed high level of resistance to antifungal agents [24]. Álvarez-Pérez et al., tested 62 *P. bovis* isolates from cases of bovine mastitis for their susceptibility to different antifungal compounds such as amphotericin B by using microdilution method [25]. All isolates were more susceptible to the polyene compounds like amphotericin B (MICs ≤ 2 μg/ml, in all cases) than other antifungals. In the present study, considering the ability of producing biofilm by *P. bovis* strains, it was evident that strains which could not produce biofilm or those which weakly produced biofilm, needed less concentration of amphotericin B to prevent their growth; while strains which showed moderate or strong biofilm production needed higher concentration of amphotericin B for their growth prevention.

Regarding the results of antimicrobial susceptibility test, the majority of strains were susceptible to gentamicin and colistin which were in agreement with other published data [8, 15, 26]. Sobukawa et al., could not find any antimicrobial drug which could prevent the growth of *Prototheca* strains in vitro [26]. Morandi et al., found that gentamicin and colistin demonstrated the highest activity against *P. zopfii* genotype 2 [8]. The investigation of antimicrobial activities of antimicrobial drugs between *P. zopfii* genotype 1 and 2 was performed using disk diffusion method by Shahid et al., [15]. *P. zopfii* genotype 2 resistance against amphotericin B and gentamicin was observed in 28.1% and 94.7% of cases. Although there is no direct relationship between in vitro antimicrobial susceptibility and natural-occurring mastitis, more in vivo trials are suggested.

## Conclusions

*P. bovis* is one of the main causative agents of bovine mastitis worldwide which causes significant economic loss. The present study showed that RAPD-PCR was a rapid and inexpensive tool for discriminating *P. bovis* strains. Also, gentamicin and colistin can be considered as potential antimicrobial drugs which can prevent the growth of the mentioned strains in vitro, although there is no effective clinical treatment yet. Thus, further studies are needed in order to detect an effective clinical therapy considering biofilm production by *Prototheca spp.* and their probable role in *Prototheca* pathogenicity.

## Methods

### Herd selection and milk samples

From Spring 2018 to Winter 2019, among all different dairy farms sending quarter milk samples from cows infected by clinical and subclinical mastitis to the laboratory under cold chain storage, 4 of them (A to D) -located in Khorasan Razavi province, Iran- were infected with *Prototheca* spp.; thus, quarter milk samples were taken from those farms and submitted to the laboratory immediately after collection and cultured for the detection of *Prototheca* spp.

One hundred µl of each milk sample was spread onto a blood agar plate and incubated aerobically at 37 °C for 48–72 h based on Shahid et al*.,* study [15]. Cell morphology of suspected colonies were assessed using wet slide and gram staining methods to investigate them under light microscope. Purification of colonies grew on blood agar was conducted using Sabouraud broth (SB). The isolates of *Prototheca spp.* were cryopreserved at − 20 °C for further analysis.

### DNA extraction and multiplex PCR

In order to extract *Prototheca* DNA, GeneAll™ extraction kit was used. To further confirm the identity of strains, all *Prototheca* isolates were subjected to the multiplex PCR described by Capra et al., [20]. Sequence of primers and their combination for multiplex PCR experiment is shown in Table 1.

**Table 1:** Name, sequence, amplicon size and specificity of all primers used in the study [20]

Primer name	Primer sequence. (5` 3`)	Amplicon size (bp)	Specificity
N476-F	TCGGAGTTAGCTGGTTCTCC	216	All *Prototheca* spp.
N476-R	ATTTTGGGGCCTTAACTGGT		All *Prototheca* spp.
N2-F	TGTAATAGATATTAGAAACGCAACAAA	508	*P. zopfii* genotype 2
N2-R	GCAGCAGTAGGGAATTTTGG		*P. zopfii* genotype 2 and *P. blaschkeae*
Bl2-F	CTTCGCCTTTGGCCTTCT	379^a^	*P. blaschkeae*
Wk3-F	CGGGAATCTTCGGATCATTA	115	*P. wickerhamii*
Wk5-R	GGTCAAATGCTTAAAGGCGTA		*P. wickerhamii*

^a^The 379 bp amplicon size is the product of combination of primers Bl2-F and N2-R

The PCR mix comprised 10 µmol L^−1^ Master mix, 3 µmol L^−1^ DNA template, 1 µmol L^−1^ for each primer and 3 µmol L^−1^ distilled water. The cycling conditions were 15 min at 94 °C, followed by 30 cycles of 1 min at 94 °C, 1 min at 60 °C, 1 min at 72 °C, and finally 7 min at 72 °C. The amplified PCR products were analyzed by electrophoresis using 1.2% (w/v) agarose gel and Green Viewer safe stain (0.01 v/v).

### RAPD-PCR and dendrogram

The RAPD-PCR (Random Amplified Polymorphism DNA-Polymerase Chain Reaction) technique was used to detect the presence of the genetic diversity of 48 *Prototheca* strains. Based on the results of RAPD-PCR assay developed by Morandi et. al, 2 primers were used in the present study [8]: OPA-4 (5′-AATCGGGCTG-3′) and OPA-18 (5′-GAGAGC- CAAC-3′). The amplification started with an initial denaturation at 94 °C for 2 min, followed by 55 cycles at 94 °C for 1 min, 40 °C for 2 min, 72 °C for 2 min, and final extension at 72 °C for 5 min.

Dendrogram and analysis of RAPD profile of strains was carried out by using GelJ software. Strains with a similarity coefficient equal to or higher than 80% may be considered to be extremely close genotypically, and perhaps even identical.

### Biofilm formation

A modification of the 96-well microtiter plate assay was applied [8]. *Prototheca* cell suspension in SB was prepared and incubated for 48 h at 37 °C (Shin et al. 2002). Next, a 96-well, flat-bottom cell culture plate was filled with 200 μL of *Prototheca* cultures diluted 1:9 in SB. Each strain was tested in triplicate. Wells with negative controls contained only SB. Plates were incubated at 37 °C, without agitation, for 24 h. Afterward the medium was aspirated and wells were washed with PBS, dried at 45 °C for 3 h, stained with 200 μL of 2% crystal violate for 20 min, rinsed with sterile water, and dried at room temperature. The stain that was bound to the biofilm was solubilized by addition of 200 μL of 33% acetic acid. Absorbance of this solution at 490 nm (OD490) was measured with microplate reader. Results were expressed as optical density (OD) values. Negative control triplicates containing only sterile SB were used as reference to determine the capacity of the *Prototheca* strains to produce biofilm. The capacity of the isolates to produce biofilm was classified as weak (OD_NC_ < OD ≤ 2 × OD_NC_), moderate (2 × OD_NC_ < OD ≤ 4 × OD_NC_), or strong (OD > 4 × OD_NC_), where OD_NC_ is the optical density of the negative control [8].

### Minimum Inhibitory Concentration (MIC)

The minimum inhibitory concentration (MIC) of amphotericin B was determined for *Prototheca* strains using E-tset strips (Tanabiotech^©^ company) and Mueller–Hinton agar plates. After 48 h of incubation at 37 °C, a symmetrical inhibition ellipse centered along the strip was formed. The MIC was read directly from the scale in terms of µg/mL, at the point where the edge of the inhibition ellipse intersected with the MIC Test Strip (µg/ml) [27].

### Antimicrobial susceptibility

The antibiotic susceptibility was determined by the disc diffusion method performed on Mueller Hinton agar according to the Clinical and Laboratory Standards Institute (CLSI, 2007 [28]). The following antimicrobial drugs were used: enrofloxacin (5 µg), Sulfonamide + Trimethoprim (25 µg), ceftriaxone (30 µg), gentamicin (10 µg), oxytetracycline (30 µg), lincomycin (2 µg), tylosin (30 µg), florfenicol (30 µg), penicillin (10 µg), colistin (10 µg), amoxicillin (25 µg), streptomycin (10 µg) and tetracycline (30 µg). Plates were incubated for 48 h at 37 °C and the diameter of growth inhibition zones was measured (mm). No universally accepted guidelines specific for *Prototheca spp.* applicable in the interpretation of drug susceptibility testing were available. According to Morandi et al*.* and based on the size of inhibition zone, the strains were divided into 3 categories: susceptible (≥ 9 mm), intermediate (3–8 mm) and resistant (≤ 2 mm) [8].

### Statistical analysis

All data were analyzed by IBM SPSS version 25. The relationship between MIC values and the ability of biofilm formation in different farms and seasons was compared using non-parametric Kruskal–Wallis test. Mann–Whitney U test was used to evaluate MIC differences within two years of sampling. Biofilm production ability of isolates in different farms were evaluated using Chi-square test.

## Data Availability

The datasets used and/or analyzed during the current study available from the corresponding author on reasonable request.

## Abbreviations

*P. bovis*: *Prototheca bovis*
MIC: Minimum Inhibitory Concentration
RAPD: Random Amplified Polymorphic DNA
OD: Optical Density
